# 

**Published:** 1890-09

**Authors:** 


### Bristol Medico-Chirurgical Journal.

---

### SEPTEMBER, 1890.

---

### CONTENTS.

Original Papers: *Page*

Epidemic Influenza. By Wm. Johnstone Fyffe, M.D. ... ... ... 147

The Bristol Infirmary in my Student Days, 1822—1828. By Henry Alford, F.R.C.S. ... ... ... ... ... ... ... ... ... ... ... 165

Clinical Records:

Clinical Studies of Disease in Children. By Eliza L. Walker Dunbar, M.D. Zürich, L.K.Q.C.P.I. ... ... ... ... ... ... ... 192

A Case of Congenital Heart-Disease. By Lockhart Stephens, M.R.C.S., L.S.A. ... ... ... ... ... ... ... ... ... ... ... 196

Reviews of Books ... ... ... ... ... ... ... ... ... ... ... ... ... ... 199

Notes on Preparations for the Sick ... ... ... ... ... ... ... ... 206

Periscope ... ... ... ... ... ... ... ... ... ... ... ... ... ... ... 211

Scraps ... ... ... ... ... ... ... ... ... ... ... ... ... ... ... ... 215

